# Heparin-binding protein in ventilator-induced lung injury

**DOI:** 10.1186/s40635-018-0198-x

**Published:** 2018-09-10

**Authors:** Jonas Tydén, N. Larsson, S. Lehtipalo, H. Herwald, M. Hultin, J. Walldén, A. F. Behndig, J. Johansson

**Affiliations:** 1 Department of Surgical and Perioperative Sciences, Anaesthesiology and Critical Care Medicine (Östersund), Umeå University, Umeå, Sweden; 2 Department of Surgical and Perioperative Sciences, Anaesthesiology and Critical Care Medicine (Umeå), Umeå University, Umeå, Sweden; 3 Department of Cell and Molecular Biology, Lund University, Lund, Sweden; 4 Department of Surgical and Perioperative Sciences, Anaesthesiology and Critical Care Medicine (Sundsvall), Umeå University, Umeå, Sweden; 5 Department of Public Health and Clinical Medicine, Division of Medicine, Umeå University, Umeå, Sweden; 6 Anestesiläkaravdelningen, Östersund Hospital, 831 32 Östersund, Sweden

**Keywords:** Ventilator-induced lung injury, HBP, Pigs, Neutrophils

## Abstract

**Background:**

Although mechanical ventilation is often lifesaving, it can also cause injury to the lungs. The lung injury is caused by not only high pressure and mechanical forces but also by inflammatory processes that are not fully understood. Heparin-binding protein (HBP), released by activated granulocytes, has been indicated as a possible mediator of increased vascular permeability in the lung injury associated with trauma and sepsis. We investigated if HBP levels were increased in the bronchoalveolar lavage fluid (BALF) or plasma in a pig model of ventilator-induced lung injury (VILI). We also investigated if HBP was present in BALF from healthy volunteers and in intubated patients in the intensive care unit (ICU).

**Methods:**

Anaesthetized pigs were randomized to receive ventilation with either tidal volumes of 8 ml/kg (controls, *n* = 6) or 20 ml/kg (VILI group, *n* = 6). Plasma and BALF samples were taken at 0, 1, 2, 4, and 6 h. In humans, HBP levels in BALF were sampled from 16 healthy volunteers and from 10 intubated patients being cared for in the ICU.

**Results:**

Plasma levels of HBP did not differ between pigs in the control and VILI groups. The median HBP levels in BALF were higher in the VILI group after 6 h of ventilation compared to those in the controls (1144 ng/ml (IQR 359–1636 ng/ml) versus 89 ng/ml (IQR 33–191 ng/ml) ng/ml, respectively, *p* = 0.02).

The median HBP level in BALF from healthy volunteers was 0.90 ng/ml (IQR 0.79–1.01 ng/ml) as compared to 1959 ng/ml (IQR 612–3306 ng/ml) from intubated ICU patients (*p* < 0.001).

**Conclusions:**

In a model of VILI in pigs, levels of HBP in BALF increased over time compared to controls, while plasma levels did not differ between the two groups.

HBP in BALF was high in intubated ICU patients in spite of the seemingly non-harmful ventilation, suggesting that inflammation from other causes might increase HBP levels.

**Electronic supplementary material:**

The online version of this article (10.1186/s40635-018-0198-x) contains supplementary material, which is available to authorized users.

## Background

Respiratory failure requiring mechanical ventilation is a common reason for admission to intensive care units (ICUs). While lifesaving, mechanical ventilation can in itself cause injury to lung tissue [1, 2].

The lung injury caused by mechanical ventilation is in part due to barotrauma and mechanotrauma, and triggering of an inflammatory process in the lungs plays an important role in worsening the injury. This is sometimes referred to as biotrauma [3]. Central parts of this inflammatory process depend on neutrophil activity, and a dominant feature of the lung injury is increased vascular permeability causing pulmonary oedema [3]. It has been shown that leukocyte depletion attenuates ventilator-induced lung injury (VILI) [4]. While it is well known that release of, for example, tumour necrosis factor alpha, interleukin 6, and interleukin 1-beta are associated with VILI, the mechanism by which leukocytes cause lung injury is not fully understood [1, 3].

Heparin-binding protein (HBP) is a protein found in the vesicles of neutrophil granulocytes. It has been shown to have several properties contributing to the inflammatory process, and its ability to increase vascular permeability in critically ill patients has been the focus of several recent studies [5–7]. Plasma levels of HBP have been found to be elevated in patients with acute respiratory distress syndrome and respiratory impairment after trauma, sepsis, and in mixed ICU patients [5, 8–10]. Increased HBP levels have also previously been shown to be associated with circulatory failure, renal failure, and mortality in critically ill patients [5–7, 10–14].

HBP levels in the bronchoalveolar fluid (BALF) have not previously been studied, and whether HBP plays a role in VILI has, to our knowledge, not been studied previously.

We hypothesized that HBP is an important mediator of leukocyte-mediated lung injury associated with harmful mechanical ventilation, and we hypothesized that levels of HBP in plasma and BALF would be elevated in a pig model of VILI. Further, we investigated if HBP was present in BALF in intubated ICU patients and in healthy volunteers, with the purpose of getting an indication as to whether the results from the animal model might be relevant in humans.

## Methods

### Animals studies

Twelve juvenile Yorkshire/Swedish landrace pigs with a mean weight of 35.5 ± 9.7 kg were used.

#### Preparation

Premedication was administered with intramuscular ketamine 10 mg/kg (Ketalar® 10 mg/ml, Pfizer AB, Sweden), xylazine 2.2 mg/kg (Rompun® vet 20 mg/ml, Bayer Animal Health, Denmark), and atropine sulphate 0.05 mg/kg (Atropine® Mylan 0.5 mg/ml, Mylan AB, Sweden). An induction dose of intravenous pentobarbital 10 mg/kg (pentobarbitalnatrium, Apoteksbolaget, Stockholm, Sweden) was given, and anaesthesia was maintained by an infusion of fentanyl 20 μg/kg/h (fentanyl, Braun, Melsungen, Germany), midazolam 0.3 mg/kg/h (Dormicum, Roche, Basel, Switzerland), and pentobarbital 5 mg/kg/h. After orotracheal intubation, the animals were mechanically ventilated (Evita 4, Dräger, Kiel, Germany) with 8 ml/kg tidal volume, ratio of inspiration/expiration (I:E) 1:2, PEEP (positive end-expiratory pressure) 5 cm H_2_O, FiO_2_ 0.4, and respiratory rate set to maintain normocapnia. Under sterile conditions, a central venous line was percutaneously inserted through the external jugular vein and an arterial line was placed in the carotid artery after a cutdown procedure. Ringer’s acetate (Ringer-Acetat® Baxter Viaflo, Baxter Medical AB, Sweden) was given at a rate of 4–5 ml/kg/h. If hypovolemia was suspected, a bolus of 250 ml of hydroxyethyl starch solution (Voluven®, Fresenius-Kabi, Uppsala, Sweden) was administered.

A warming gel pad was used to maintain a steady body temperature of 38.0 °C, as measured per rectum.

#### Protocol

To render the animals’ lungs more susceptible to VILI, surfactant depletion was performed by saline lavage in all animals. After preoxygenation with FiO_2_ 1.0, 30 ml/kg of 38 °C 0.9% sterile normal saline was instilled into the trachea and then allowed to drain passively through the tracheal tube. The procedure was repeated a total of four times, interspaced by a short recovery period to allow the arterial oxygen saturation to return to at least 96%.

**Fig. 1:**
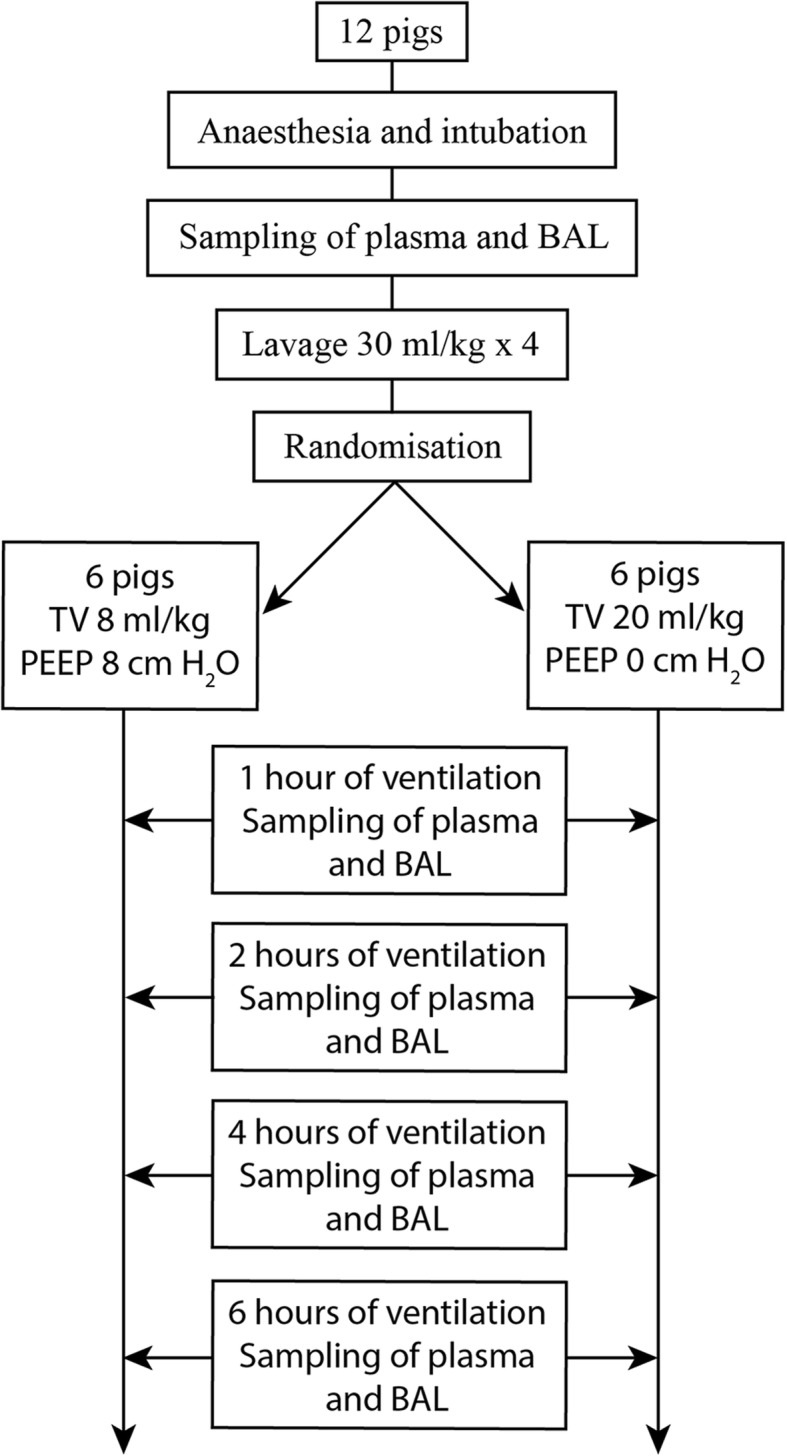
Schematic overview of the protocol. BAL bronchoalveolar lavage, TV tidal volume, PEEP positive end-expiratory pressure

Immediately after surfactant depletion, the animals were randomized to either protective or injurious ventilation. In the group with protective ventilation, tidal volumes were kept at 8 ml/kg with I:E 1:2 and PEEP was elevated to 8 cm H_2_0. Respiratory rate was adjusted to normocapnia, and FiO_2_ was set to achieve normoxia. The group with injurious ventilation (referred to as the VILI group) received tidal volumes of 20 ml/kg with I:E 1:2, respiratory rate 20 bpm, ZEEP (zero end-expiratory pressure), and FiO_2_ 1.0. Extra tubing was added as needed between the endotracheal tube and the Y-piece to increase the dead space to aim for normocapnia. The animals were ventilated in either fashion for 6 h and then euthanized by injection of potassium chloride under continuous deep anaesthesia. Samples of blood and BALF were collected before surfactant depletion and after 1, 2, 4, and 6 h of ventilation (Fig. 1).

Biopsies from the upper and lower lobes of both lungs were collected during autopsy. The histology of the biopsies was examined by a pathologist, blinded to group assignment, evaluating acute lung injury looking at oedema, bleeding, membranes, alveolar inflammation, and bronchiolitis. The severity of lung injury was graded as no, mild, moderate, or severe.

#### Sampling procedures

BALF was collected using a flexible fiberoptic bronchoscope. The bronchoscope was inserted through the tracheal tube and carefully wedged into a bronchus. As sampling was repeated, care was taken to avoid multiple samples from the same location. Three aliquots of 50 ml sterile saline were infused and gently suctioned back after each infusion. The BALF was recovered in a glass container placed in ice water. After immediate filtration through a 100-μm nylon filter, the recovered volume was noted and the BALF was centrifuged for 15 min at 4 **°**C at 400 RCF. The supernatants were separated from the cell pellet, divided into aliquots, and immediately frozen at − 70 **°**C. The cell pellets were resuspended in phosphate-buffered saline at a cell concentration of 10^6^ cells/mL for differential cell counts.

Blood samples were collected from the arterial line using standard EDTA vacutainer tubes. The blood samples were immediately put on ice and centrifuged for 10 min at 4 **°**C at 2000 RCF. The plasma was then recovered and immediately frozen in aliquots at − 70 **°**C.

### Human studies

#### Healthy volunteers

Sixteen healthy, non-smoking subjects were recruited to the study.

The subjects received 1 mg atropine subcutaneously as pre-medication. Lidocaine was used for topical anaesthesia. Bronchoscopy was performed using a flexible video bronchoscope (Olympus BF IT200; Olympus, Tokyo, Japan) introduced through the mouth. Bronchoalveolar lavage (BAL) with 3 × 60 ml of saline solution from the middle or lingula lobes was performed. The recovered aspirate was filtered to eliminate mucus (pore diameter 100 μm) and centrifuged at 400×*g* for 15 min to separate the cellular components from the supernatant, which was stored at − 80 °C until analysis.

#### ICU patients

Ten patients older than 18 years of age admitted to the ICU of Östersund Hospital and intubated during the previous 24-h period were included.

The SAPS 3 at admission to the ICU and the highest respiratory pressure settings from intubation to sampling were recorded. Plasma levels of HBP were sampled at the time of intubation. Diagnosis was determined retrospectively through chart review.

BALF was collected using a flexible fiberoptic bronchoscope. The bronchoscope was inserted through the tracheal tube and wedged into a bronchus of the middle or lingual lobe. If lung pathology was unilateral, the contralateral side was used. A total volume of 50 ml of sterile saline was instilled and suctioned back four times. The obtained fluid was filtered and centrifuged at 400×*g*, and the supernatant was frozen at − 80 °C until analysis.

### Analysis of HBP

Concentrations of HBP in the samples were measured as described previously [15]. In short, a sandwich ELISA with a monoclonal and polyclonal antibody against pig or human HBP was used to measure the concentrations. The technique has an intra- and inter-assay variability of less than 10%.

### Statistics

Descriptive data are presented as medians with interquartile ranges. Because the data were not normally distributed, the Mann–Whitney *U* test or related samples Wilcoxon signed-rank test was used for comparison between groups as appropriate. The significance of differences between categorical variables was evaluated using Fisher’s exact test. Probabilities of less than 0.05 were accepted as significant. The data were analysed using SPSS (IBM SPSS Statistics for Macintosh, Version 23.0, Armonk, NY: IBM Corp).

## Results

### Animal studies

As intended, the VILI group received significantly higher FiO_2_, larger tidal volumes, and higher inspiratory pressures and had lower respiratory rates compared to the control group. Oxygenation was significantly impaired in the VILI group. Arterial carbon dioxide levels were not significantly different between the groups, whereas the pH was lower in the VILI group. The average total fluid administration per hour was higher in the VILI group. There were two premature deaths during the experiment in the VILI group, and none in the control group. Other baseline characteristics and physiological measurements were similar between the two groups (Table 1).

**Table 1:** Characteristics of the pigs by study group. Data are presented as the median (interquartile range)

	Control *n* = 6	VILI *n* = 6	*p* value
Weight (kg)	35.5 (28–50)	35.5 (29–47)	0.818
Premature death (*n*)	0	2	
Tidal volume (ml)	280 (228–400)	710 (575–940)	0.002*
Respiratory rate (breaths/min)	30 (26–38)	20 (20–20)	0.002*
Average peak inspiratory pressure (cmH_2_O)	22 (20–23)	43(39–48)	0.002*
FiO_2_	0.35 (0.29–0.40)	1.0 (1.0–1.0)	0.002*
Worst P/F ratio (kPa)	46 (34–52)	14 (8–39)	0.041*
Last P/F ratio	53 (43–64)	25 (8–43)	0.065
Last PCO_2_ (kPa)	4.6 (3.8–5.0)	5.9 (4.7–7.0)	0.093
Last pH	7.52 (7.47–7.55)	7.41 (7.31–7.48)	0.026*
Total crystalloid (ml)	2000 (975–3000)	2500 (1800–3200)	0.394
Total colloid (ml)	0 (0–500)	375 (0–500)	0.485
Average fluid admin. (ml/kg/h)	7.7 (4.4–8.2)	10.0 (8.5–12.0)	0.026*
Average CVP (mmHg)	8 (7–8)	7 (6–9)	0.394
Average MAP (mmHg)	70 (67–75)	75 (53–102)	0.24
Average heart rate (bpm)	87 (82–95)	103 (94–111)	0.065
Average temp (°C)	39.4 (38.3–39.7)	38.5 (38.0–39.3)	0.394
Recovered BALF volume (%)	78 (62.5–84.0)	71 (60.0–79.0)	0.168

*FiO*_*2*_ fraction of oxygen in inspired gas, *P/F ratio* ratio between partial pressure of oxygen in arterial blood (kPa) and simultaneous FiO_2_, *PCO*_*2*_ partial pressure of carbon dioxide in arterial blood, *CVP* central venous pressure, *MAP* mean arterial pressure, *BALF* bronchoalveolar lavage fluid

Asterisks indicate statistical significance (*p* < 0.05)

In the VILI group, 2 biopsies were lost or damaged. Out of the remaining 34, none showed no lung injury, 1 showed mild lung injury, 10 showed moderate lung injury, and 23 showed severe lung injury. In the control group, 5 biopsies were lost or damaged. Out of the remaining 24, 14 showed no lung injury, 4 showed mild lung injury, 1 showed moderate lung injury, and none showed severe lung injury.

There were significantly more biopsies showing severe (*p* < 0.01) and moderate (*p* = 0.047) lung injury in the VILI group. There were significantly more biopsies showing no lung injury (*p* < 0.01) in the control group.

**Fig. 2:**
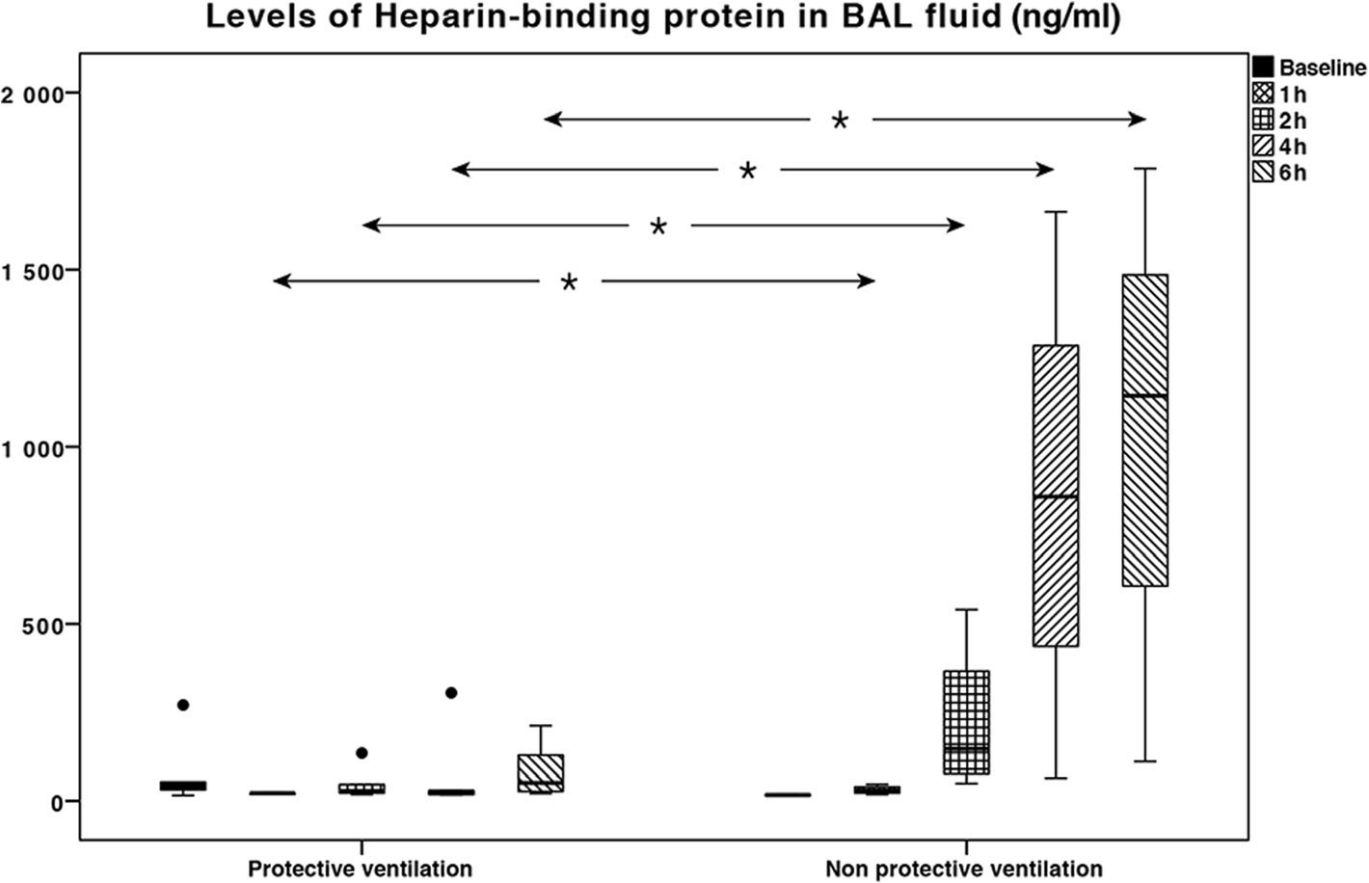
Levels of heparin-binding protein (HBP) (ng/ml) in the bronchoalveolar lavage (BAL) fluid from pigs. HBP levels were significantly higher in the group receiving harmful ventilation at 2 h (*p* = 0.03), 4 h (*p* < 0.01), and 6 h (*p* = 0.02) of ventilation. Boxes indicate the second to third quartile with the median. Brackets indicate min-max values, circles indicate outliers, and stars indicate significant differences (*p* < 0.05)

HBP levels in plasma did not differ significantly between the groups at any time of sampling (Table 2). HBP levels in BALF were significantly higher in the VILI group after 1 h (*p* = 0.04), 2 h (*p* = 0.03), 4 h (*p* < 0.01), and 6 h (*p* = 0.02) of ventilation (Table 2, Fig. 2).

**Table 2:** Levels of heparin-binding protein and neutrophil counts in pigs subjected to injurious ventilation and controls

Baseline	HBP in BALF (ng/ml)			HBP in plasma (ng/ml)			Neutrophil count in BALF (×10^4^/ml)		
	Control	VILI	*p*	Control	VILI	*p*	Control	VILI	*p*
	45 (32–216)	17 (14–18)	0.01	23 (16–29)	21 (20–25)	0.84	1.1 (0.5–2.0)	0.9 (0.2–1.4)	0.49
1 h	20 (18–23)	29 (20–43)	0.04*	31 (21–58)	23 (19–34)	0.70	1.0 (0.7–1.2)	1.4 (0.8–2.5)	0.39
2 h	25 (19–42)	147 (62–453)	0.03*	25 (18–31)	24 (18–36)	0.69	1.3 (0.6–2.2)	6.3 (4.6–12.6)	< 0.01*
4 h	22 (17–235)	859 (250–1475)	< 0.01*	28 (24–39)	35 (23–39)	0.94	1.7 (0.8–4.1)	22.1 (6.6–28.2)	0.05
6 h	89 (33–191)	1144 (359–1636)	0.02*	33 (31–44)	61 (47–77)	0.13	2.7 (2.2–2.8)	21.0 (6.1–33.7)	0.03*

Data are presented as the median (interquartile range). Asterisks indicate statistical significance (*p* < 0.05)

*HBP* heparin-binding protein, *BALF* bronchoalveolar lavage fluid, *VILI* ventilator-induced lung injury

In the VILI group, comparing first and last samples, plasma levels of HBP did not increase over time (*p* = 0.07) whereas HBP levels in BALF increased significantly (*p* = 0.04).

**Fig. 3:**
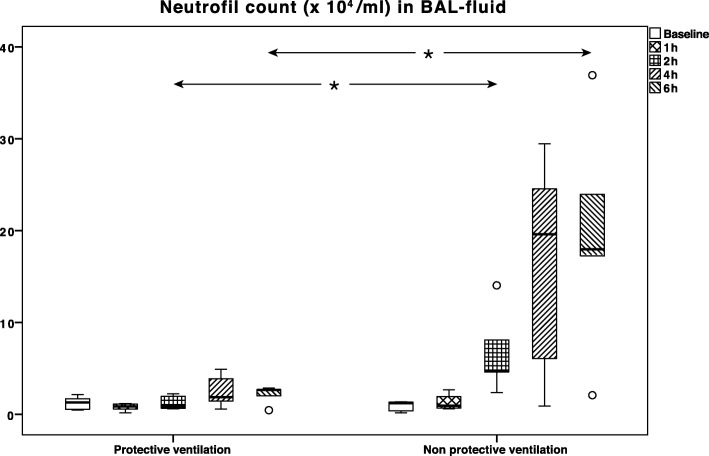
Neutrophil count (× 10^4^/ml) in the bronchoalveolar lavage (BAL) fluid. The neutrophil count was significantly higher in the group receiving harmful ventilation at 2 h (*p* < 0.01) and 6 h (*p* = 0.03) of ventilation. Boxes indicate the second to third quartile with the median. Brackets indicate the min-max values, circles indicate outliers, and stars indicate significant differences (*p* < 0.05)

The neutrophil count in BALF was significantly higher in the VILI group at 2 h (*p* < 0.01) and 6 h (*p* = 0.03) of ventilation (Table 2, Fig. 3).

### Human studies in healthy volunteers and ICU patients

The diagnoses and respiratory settings of the ICU patients are shown in Table 3. The median HBP level in plasma from the ICU patients was 31.3 ng/ml (IQR 22.4–55.5).

**Table 3:** Characteristics and levels of HBP in BALF and plasma in intubated ICU patients

Diagnosis	SAPS 3	FiO_2_ (%)	Peak pressure (cm H_2_O)	PEEP (cm H_2_O)	Tidal volume (ml/kg)	HBP level in BALF (ng/ml)	HBP level in plasma (ng/ml)
Pneumococcal sepsis	76	50	26	9	6.3	25	1220
Aspiration pneumonia	65	35	15	8	6.3	1098	23
COPD, pneumonia	91	40	22	9	5.6	1614	17
Staphylococcal pneumonia	83	55	28	11	6.1	1341	24
Fasciitis in extremity	61	45	26	12	6.3	4558	82
Status epilepticus	65	30	14	5	5.6	3254	29
Spinal trauma, delirium	53	35	20	10	7.4	2325	47
Pneumonia	56	50	24	12	5.5	4646	43
Aspiration pneumonia	51	60	17	8	6.8	2303	13
Pneumonia	68	35	15	7	5.9	251	34

*SAPS 3* Simplified Acute Physiology Score 3, *FiO*_*2*_ fraction of oxygen in inspired gas, *PEEP* positive end-expiratory pressure, *BALF* bronchoalveolar lavage fluid, *HBP* heparin-binding protein, *COPD* chronic obstructive pulmonary disease

The median HBP level in BALF from healthy volunteers was 0.90 ng/ml (IQR 0.79–1.01), and the median HBP level in BALF in intubated ICU patients was 1959 ng/ml (IQR 612–3306). The HBP levels in BALF were significantly higher in the ICU patients (*p* < 0.01).

## Discussion

In this study with data from animals and humans, we show that in pigs subjected to VILI, levels of HBP in BALF increased significantly over time, while HBP levels in plasma did not. Human healthy volunteers had low HBP levels in BALF, whereas HBP levels were significantly higher in intubated ICU patients who did not have documented VILI.

The findings imply local release of HBP in the alveoli, thus suggesting a potential pathophysiological contributor in VILI. In pigs subjected to injurious ventilation, the increased number of BALF neutrophils is in line with previous research [1]. Although the presence of several inflammatory mediators in BALF in VILI has been found previously [3], this is, to the best of our knowledge, the first time that levels of HBP have been investigated in this context.

It is highly likely that the HBP found in BALF originates from neutrophils and that the higher levels in the VILI group might simply be related to the finding of more neutrophils in this group. The higher FiO_2_ in the VILI group might have activated neutrophils and contributed to higher HBP levels.

In the ICU patients (*n* = 10), HBP levels in BALF were higher compared to the healthy volunteers. This was despite the seemingly non-injurious ventilation. This is probably due to inflammatory processes in the lungs from a variety of causes other than VILI, and thus measuring HBP levels in BALF does not seem to be a way of detecting injurious ventilation in ICU patients.

The plasma levels of HBP in the ICU patients were elevated to a level consistent with previous research on ICU patients with respiratory or circulatory failure [5, 6, 9, 16, 17].

In a recent publication, heparin was found to counteract HBP’s ability to increase vascular permeability in vitro [5]. This, together with several publications on heparin inhalation in acute lung injury with mixed results [18–20], points towards a possible way to attenuate lung injury caused by harmful ventilation. This would be an interesting topic for future studies.

Increased extravasation of granulocytes in the alveoli and increased pulmonary vascular permeability are hallmarks of ARDS. The concentrations of HBP found in BALF in ventilated patients in the ICU were very high in comparison with concentrations found in plasma, despite being diluted in the BAL process. Thus, the actual concentration in the alveoli of these patients must have been extremely high, likely by a factor of at least one thousand above what was found in plasma in the same patients. The concentrations of HBP previously used in vitro to affect vascular permeability were in this same range [21]; thus, it is likely that the patients that we studied had increased vascular permeability due to the levels of HBP that were found. Further, in a state of increased vascular permeability, it might be possible for HBP to pass the epithelial and the endothelial membranes. This means that the lungs might indeed be a source of systemic HBP in critical illness.

The pig model we used to study VILI has strengths and weaknesses. Surfactant depletion is rarely clinically relevant in adult critical care. However, since both the control and treatment groups are subjected to lavage and the groups separate well with regard to histology and HBP levels, we believe the results are relevant.

One of the pigs in the group receiving high tidal volumes did not develop histological lung injury nor did it have elevated levels of HBP in plasma or BALF or increased numbers of neutrophils compared to the control group. This is highly interesting, but we do not know why this pig was seemingly resistant to the injurious effects of the high tidal volume ventilation.

## Conclusions

In a model of VILI in pigs, the levels of HBP in BALF increased significantly over time compared to controls. Plasma levels, however, did not differ significantly between the two groups.

HBP in BAL fluid was high in intubated ICU patients in spite of the seemingly non-harmful ventilation, suggesting that inflammation from other causes might increase HBP levels.

## Additional file

Additional file 1: Datasets. Datasets of ICU patients, pigs and healthy volunteers. (XLSX 13 kb)

## Abbreviations

BALF: Bronchoalveolar lavage fluid
HBP: Heparin-binding protein
ICU: Intensive care unit
VILI: Ventilator-induced lung injury
